# Executive failure hypothesis explains the trait-level association between motivation and mind wandering

**DOI:** 10.1038/s41598-022-09824-3

**Published:** 2022-04-07

**Authors:** Toshikazu Kawagoe

**Affiliations:** School of Humanities and Science, Kyushu Campus, Tokai University, Higashi-Ku, Toroku 9-1-1, Kumamoto, 862-8652 Japan

**Keywords:** Psychology, Human behaviour

## Abstract

Mind wandering (MW) is commonly observable in daily life. Early studies established an association between motivation and MW at the trait level using a questionnaire survey. Considering that the mechanism of state-level association between them is known, this study was conducted to replicate the trait-level association and determine its possible mechanisms. Two independent samples were analysed using several questionnaires, which included motivation and MW. General one- and multi-dimensional scales were administered for both variables. Besides the successful replication of the significant association between motivation and MW at the trait level, we found that people with low levels of executive function experience high rates of spontaneous MW. This finding indicates that the underlying mechanism of trait-level association is the executive failure hypothesis, which postulates that a failure of executive control during task-related objectives evokes MW. Further, the motivation–MW relationship exhibits a different psychological basis at the state and trait levels.

## Introduction

Mind wandering (MW) is an experience wherein the mind drifts away from the task at hand towards unrelated inner thoughts, which may occur for 50% of the waking time^1^. In contrast to traditional cognitive psychological research, the MW concept focuses on subjective internal thoughts and feelings unrelated to external tasks and on the shift between them. This study uses MW as a general umbrella term, although it may include task-unrelated, stimulus-independent and self-generated thoughts^2–5^.

In the literature, motivation has attracted attention because of its association with MW. Previous studies have found a significant association between low levels of motivation and the occurrence of MW during task execution^6–8^. The intentionality of MW can explain the underlying mechanism of this state-level association as follows^9,10^. Experimental psychologists predict that participants would be moderately motivated to complete a laboratory task; however, in reality, participants may not be motivated towards performing the task. Instead, representative tasks used in MW studies are intended to demotivate participants. In these trivial, boring tasks, participants became unmotivated, thus causing their minds to wander deliberately^6,7,11^. Similarly a redundant encoding of information (i.e. re-reading), which may be uneventful and demotivating, causes deliberate MW but not spontaneous MW^12^.

In contrast to this *state*-level finding, Kawagoe et al.^13^ verified the aforementioned relationship at the depositional and/or temperament *trait* level, where individuals with low levels of trait motivation were found to experience MW more regularly in their daily life. However, the psychological mechanism underlying this trait-level association remains unknown. Thus, the current study investigated whether this psychological mechanism may apply to the trait-level association between motivation and MW. One possibility is that humans tend to intentionally let their minds wander when confronted with boring tasks and/or situations in daily life (i.e. trait level), as in the case of state level.

A variable that possibly promotes the association between motivation and MW is executive function. Throughout the process of uncovering the phenomenological characteristics of MW, scholars have investigated its association with other psychological features, such as attentional/executive control, meta-cognition and problem solving, finding that such a higher-order cognitive function is significantly correlated with MW^4,5,14^, although the directionality of the associations varies from negative^3,6,15^ to positive^2,4^, depending on the context wherein MW is measured^7^. For example, the executive function works to constrain attention only to task-relevant information in a demanding situation and may result in a negative association, while in an undemanding external task, individuals with greater cognitive ability could let their mind wander to task-irrelevant information in an adaptive manner that may cause a positive association. In trait level, accordingly, executive function could exhibit both ways of association with MW because there are tons of tasks with various degrees of difficulties in daily life, which is still unknown. It is reasonable to inspect the motivation–MW association via an index of executive function. Simultaneously, a deterioration in executive function can cause a clinical level of amotivation^16–18^, whose association can be observed in healthy individuals^19,20^.

This study views motivation as a continuum to the clinical state of *apathy*. In clinical populations, people who are consistently unmotivated are deemed apathetic, defined as the lack of motivation that is not attributable to a diminished level of consciousness, cognitive impairment or emotional distress^16,17^. As per the definition, this concept could be applied to *healthy* individuals^19,20^, especially when a comprehensive, simplified scale to assess general motivation in daily life is lacking. This is because traditionally, motivation has been understood as a *state* that is assessed in many fields, including psychology, during specific situations using explicit goals, such as provocation using various forms of incentives (for a review, see Braver et al.^21^). Previous clinical studies have indicated that apathy is multi-dimensional and can be grouped as executive, emotional and initiation-related types^17,18^. For example, the executive type of apathy refers to amotivation for planning, organisation or attention, which is apparently owing to the deterioration of executive function. Thus, we utilised the dimensional apathy scale (DAS), which comprises the executive (dasEx), emotional (dasEm) and behavioural/cognitive initiation (dasIni) sub-factors of apathy^18^. This study aims to investigate whether the executive type of amotivation is specifically associated with MW. Hereafter, we use the term *(a)motivation* instead of *apathy* in order to not focus on the participants’ clinical aspects.

In summary, the current study aims to uncover the psychological mechanism underlying the trait-level association by considering the intentionality of MW and executive function. If the trait-level association between motivation and MW depends on the same mechanism for the state level, intentionality should affect the relationship wherein a deliberate type of MW has a stronger relationship with motivation than spontaneous MW. Alternatively, if the executive control is the key, the measurements related to executive function would mediate the motivation–MW relationship. To these ends, Study 1 first intends to determine whether replicating the significant association between motivation and MW at the trait level is confirmed by using an online survey. Further, this study adopts several mediation models besides simple correlation analyses wherein each component (i.e. spontaneous/deliberate components of MW, executive component of motivation and both) mediates the association between motivation and MW in general. Notably, although mediation analysis is used in this study, we could not assume the causality among the variables. We could only expect that the covariance between motivation and executive function could cause MW. In Study 2, another independent sample was used with the objective of conducting self-replication within the study to enhance the reliability of the findings in Study 1 (in which the executive function was assessed using a specialised measurement), that is, the effortful control scale (ECS)^22,23^ was used to promote the concept that executive function mediates the relationship between motivation and MW. Specifically, this study conducted mediation analyses to verify whether executive control (i.e. ECS) mediates the effect of motivation on MW.

## Study 1

### Participants

This study used an online survey. All participants were recruited via iBRIDGE Corporation, a data collection company, and are assumed to be Japanese based on their location. They were compensated by a small amount of ‘points’ set by the data collection company. One thousand participants (aged 20–69 years) were recruited with the objective of including 200 participants in every age group (i.e. 20–29, 30–39, and so on), which resulted in 1009 participants in total. Informed consent was obtained from all participants. The appropriate ethics committee approved the study, which was conducted in accordance with the Declaration of Helsinki (1975, as revised in 2008).

Three *lure* items (i.e. ‘Please select the most correct choice for this item’) were added to identify participants who answered without paying sufficient attention to the items. The study was concerned about a possible *satisficing* behaviour from the participants, which may contaminate the data^24^. Participants who failed to follow the instructions were excluded from the analyses. Finally, data from 587 participants were analysed (mean age = 47.3 ± 13.5 years; women = 301). The sample size met the rule of thumb for regression analysis, that is, 100 participants + 100 per predictor variable^25,26^.

### Measurements

This study investigated the level of motivation (or apathy) and MW at the trait level. Two questionnaires were used to assess motivation and MW, namely, a one-dimensional scale and a multi-dimensional one.

#### Scales for motivation

##### Apathy scale (AS)

AS is a 14-item questionnaire that asks participants if they have experienced a lack of motivation^27^. Each item is scored from 0 to 3; the total scores range from 0 to 42, with higher scores indicating increased amotivation. AS is used worldwide, and it exhibits good validity and reliability. This study employed the Japanese version of the scale^28^. Sample items include the following: ‘Are you interested in learning new things?’ and ‘Do you have the energy for daily activities?’

##### Dimensional apathy scale (DAS)

DAS is used to assess apathy in a multi-dimensional manner^18^, using three dimensions, namely, executive, emotional and initiation, which are based on rigorous clinical and neurological observations^17^. Each dimension is assessed using eight items, with scores ranging from 0 to 3. In total, the sub-scores ranged from 0 to 24 for each dimension, with higher scores indicating higher levels of amotivation, which is similar to what holds for AS. We utilised the Japanese version of the scale^29^. Sample items for the executive, emotional and initiation subscales are as follows: ‘I find it hard to concentrate on things’, ‘Before I do something, I think about what others would feel about it’ and ‘I contact my friends’.

#### Scales for mind wandering

##### Mind wandering questionnaire (MWQ)

MWQ is a single-factor questionnaire that assesses an individual’s tendency towards MW^30^. MWQ comprises five items rated using a 6-point Likert-type scale. The total scores ranged from 5 to 50, with higher scores indicating higher levels of MW tendency. This study used the Japanese version of the scale^31^. A sample item is as follows: ‘I have difficulty maintaining focus on simple or repetitive work’.

##### Mind wandering: deliberate (MW-D) and spontaneous (MW-S)

MW-D and MW-S were used to assess MW with and without intention, respectively^32^. Each scale comprises four items rated using a 7-point Likert-type scale. Scores ranged from 4 to 28, with higher scores indicating increased MW tendency for each type. The Japanese versions of the scale were used^33^. Sample items include the following: ‘I allow my thoughts to wander on purpose’ (MW-D) and ‘I find my thoughts wandering spontaneously’ (MW-S).

### Analyses

This study primarily used correlational analyses. The 95% confidence intervals (CIs) for each correlation coefficient were listed. Although correlational analyses were conducted in a sequential manner, multiplicity was considered by using the Bonferroni correction given the number of possible combinations to strengthen the reliability of the results. To investigate the specificity of these associations, Fisher’s *r* to *z* transformation was used to statistically compare the correlation coefficients. Although skewness and kurtosis were within acceptable ranges (< |2|)^34^ (Tables 1 and 2), MW-D and MW-S seemingly intruded on the distribution of normality with a floor effect (Figs. S1 and S2). Therefore, supplemental non-parametric analyses were implemented for the correlations that included the two variables. Moreover, based on the results of the correlation, several mediation analyses were conducted to elucidate the structure of the current data. To investigate whether the sub-components (executive for motivation and spontaneous for MW) mediate the association between motivation and MW in general, the study set the independent and dependent variables as AS and MWQ, respectively. In Models 1 and 2, the mediators were MW-S and dasEx, respectively. In Model 3, MW-S and dasEx were simultaneously included. Besides the conventional *z*-test, bootstrapping (2,000 samples) was used to test the significance of the indirect effects, which does not require assuming normality of the sample distribution^35^. These non-nested models were compared via Vuong’s likelihood ratio tests^36,37^. The analyses and visualisation were performed using R (https://www.R-project.org/).

**Table 1:** Descriptive information of study 1 (N = 587).

Measure	Mean	SD	Skewness	Kurtosis	Cronbach’s alpha
AS	16.94	6.98	0	0.17	0.87
DAS-executive	7.99	4.32	0.45	− 0.05	0.82
DAS-emotional	11.84	2.88	− 0.20	0.58	0.41
DAS-initiation	15.07	4.45	− 0.49	0.39	0.83
MWQ	14.44	5.03	0.12	0	0.84
MW-D	11.45	5.42	0.22	− 0.78	0.85
MW-S	11.41	6.15	0.41	− 0.79	0.95

*AS* apathy scale, *DAS* dimensional apathy scale, *MWQ* mind wandering questionnaire, *MW-D* deliberate mind wandering scale, *MW-S* spontaneous mind wandering scale, *SD* standard deviation.

**Table 2:** Descriptive information of study 2 (N = 562).

Measure	Mean	SD	Skewness	Kurtosis	Cronbach’s alpha
AS	17.85	6.86	− 0.15	− 0.12	0.85
DAS-executive	8.47	4.07	0.5	0.16	0.80
DAS-emotional	11.73	3.00	− 0.42	0.57	0.50
DAS-initiation	15.42	4.04	− 0.37	0.22	0.79
MWQ	14.71	4.96	− 0.15	− 0.35	0.80
MW-D	10.92	4.89	0.21	− 0.69	0.82
MW-S	10.77	5.71	0.49	− 0.56	0.92
ECS	97.25	14.43	0.43	0.1	0.91

*AS* apathy scale, *DAS* dimensional apathy scale, *MWQ* mind wandering questionnaire, *MW-D* deliberate mind wandering scale, *MW-S* spontaneous mind wandering scale, *SD* standard deviation and *ECS* effortful control scale.

### Results

Table 1 presents the descriptive information of the participants, which is visualised in Fig. S1. First, to replicate a previous report of the significant association between motivation and MW at the trait level^13^, this study employed correlation analysis and found that the replication was successful, where the lower the motivation, the higher the MW rates experienced at the trait level (AS-MWQ: *r* = 0.39 [95% CI 0.32–0.46], *p* < 0.001). Figure S3 presents the correlation coefficients among the variables.

**Figure 1:**
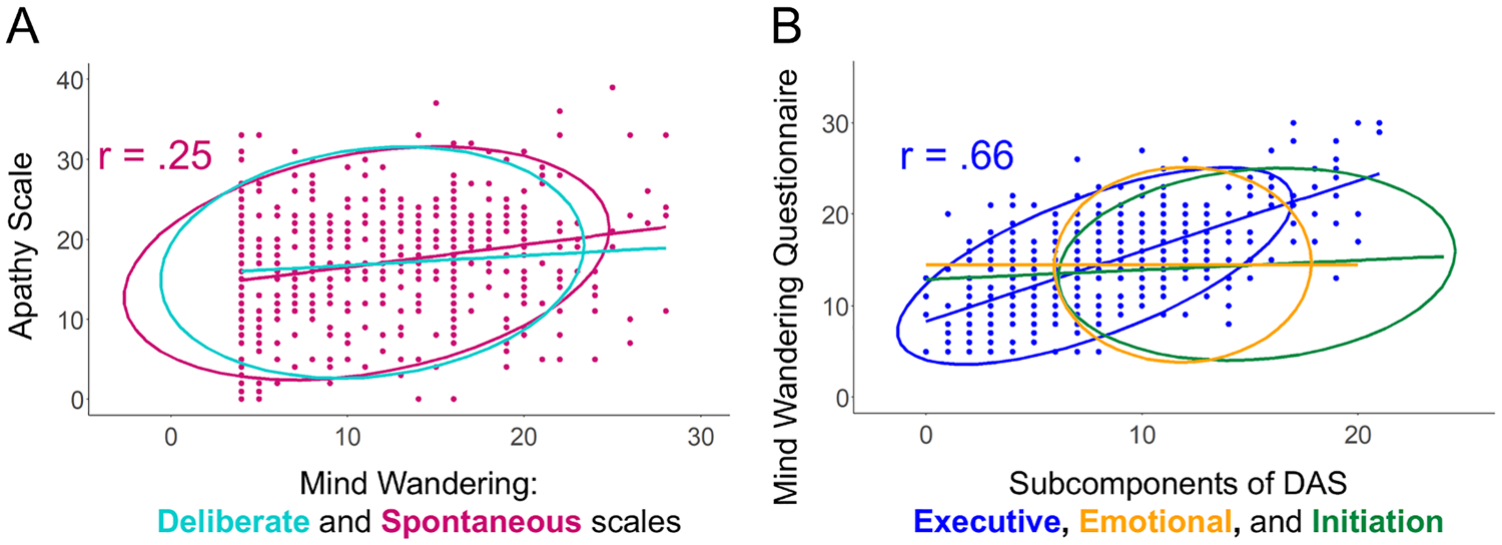
Scatter Plots of the Correlations Between Mind Wandering and Motivational Indices in Study 1. *Note* Data for non-significant correlations after multiple corrections are only shown by the 95% elliptical confidence region of the multivariate t-distribution. DAS = dimensional apathy scale. Higher scores on the apathy scale and DAS indicate increased amotivation.

To test the hypothesis that people who are unmotivated intentionally allow their minds to wander, this study assessed the correlations between AS and MW-D/S. A significant correlation was found between AS and MW-S (*r* = 0.25 [95% CI 0.18–0.33], *p* < 0.001) but not between AS and MW-D (*r* = 0.10 [95% CI 0.02–0.18], *p* = 0.248) (Fig. 1A). These correlation coefficients differed significantly (*z* = 4.69, *p* < 0.001). The second hypothesis, that is, the executive component will modulate the relationship between motivation and MW, was also tested. MWQ was significantly correlated with dasEx (*r* = 0.66 [95% CI 0.61–0.70], *p* < 0.001) but not with the other sub-components of DAS (dasEm: *r* = 0.00 [95% CI − 0.08 to 0.08], *p* = 0.988; dasIni: *r* = 0.09 [95% CI 0.01–0.17], *p* = 0.522) (Fig. 1B). This difference was also significant (*z*s > 12.3, *p*s < 0.001). As an additional analysis, non-parametric correlational analysis was conducted for measures whose distributions violated normality. This analysis did not influence the significance of the results, as shown in the Supplementary Material.

**Figure 2:**
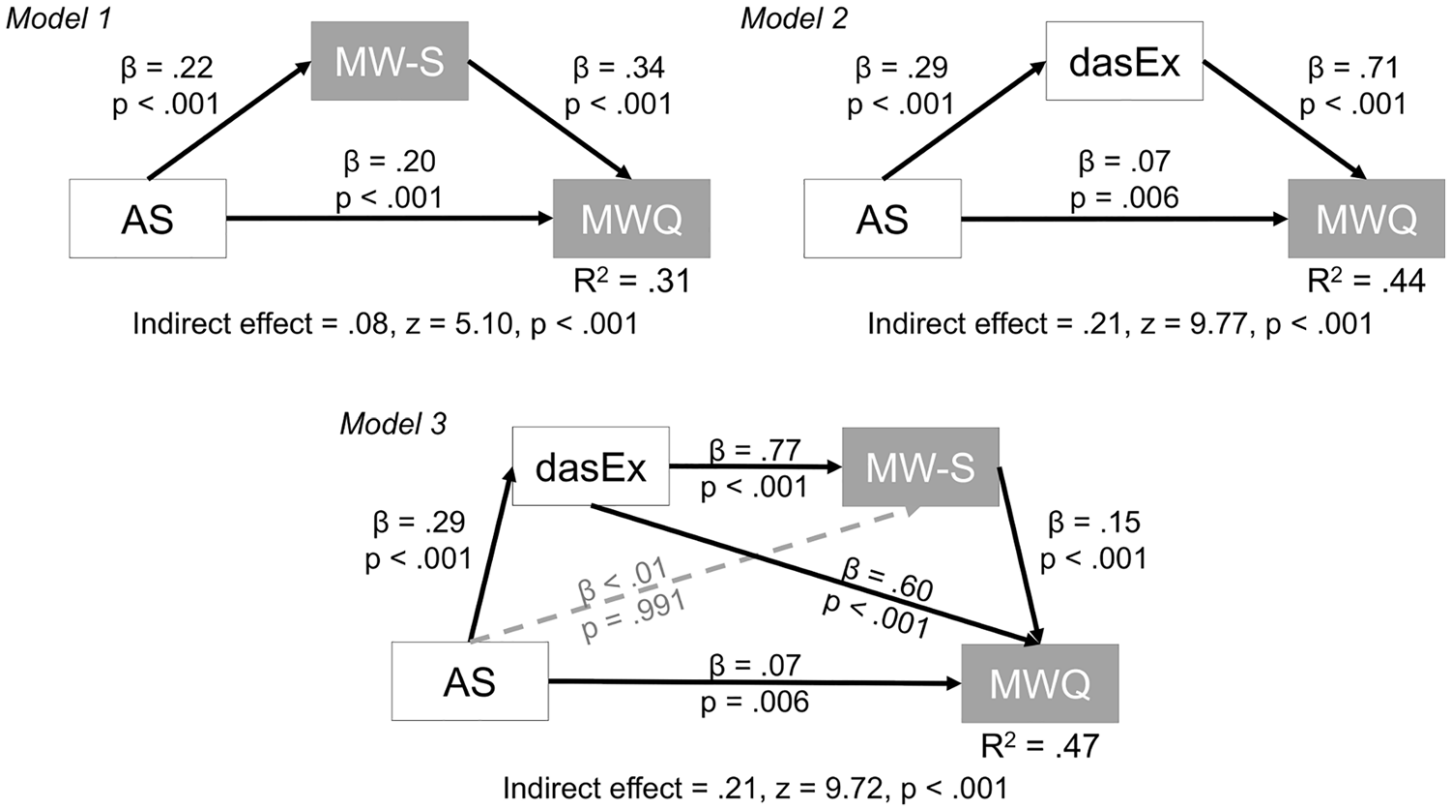
Mediation Analyses of the Relationship Between the Apathy Scale and Mind Wandering Questionnaire. *Note* Two mediators were considered, namely, the spontaneous mind wandering scale (Model 1) and executive subscale of DAS (Model 2). Model 3 incorporated them simultaneously. AS = apathy scale; MW-S = spontaneous MW scale, MWQ = mind wandering questionnaire; DAS = dimensional apathy scale and dasEx = executive subcomponent of DAS.

These results indicated that the executive component of motivation and/or spontaneous MW mediates the relationship between motivation and MW. Additional mediation analyses were conducted using several models. Figure 2 presents the graphical results. In Models 1 and 2, the indirect effect was significant, indicating that the two variables significantly functioned as a mediator in the association between AS and MWQ. A comparison via the robust likelihood ratio test between the two non-nested models suggested that Model 2 is superior to Model 1 (robust likelihood ratio = 644, *p* < 0.001). When the variables were included simultaneously, the indirect effect was again significant (Model 3). However, Model 3 fit worse than Model 2 (robust likelihood ratio = 2924, *p* < 0.001). The bias-corrected bootstrapped CIs for indirect effects were above 0 in all models (0.05–0.11 in Model 1, 0.17–0.25 in Model 2 and 0.17–0.25 in Model 3).

## Interim discussion

A person’s MW tendency is related to several factors^1,5,14^. Intuitively, motivation towards tasks influences the rate of MW during such tasks^6–8,11^. A previous study also identified this association between motivation and MW at the trait level^13^. However, the underlying mechanism remains unclear.

The significance of trait-level association between motivation and MW could be replicated in Study 1 using a different sample based on a previous report^13^, which indicated that the association is robust in healthy individuals. This finding is important because this relationship was not intuitive at the trait level. Next, simple correlation analyses indicated that the executive component of motivation and the spontaneous component of MW contribute to the association. The following analyses supported the possibility that these sub-components mediate the association between motivation and MW. Statistically, the executive type of motivation and spontaneous MW partially mediated this association. Their mediating role can also be observed in the model that simultaneously posits the two variables as mediators (i.e. Model 3), although Model 2 displayed superiority in terms of parsimoniousness. Importantly, the result of Model 3 provides knowledge that dasEx entirely mediates the effect of AS on MW-S, whereas MW-S significantly mediates the relationship between dasEx and MWQ. This outline is reminiscent of the executive failure hypothesis of MW^3,15^. As this hypothesis is in accordance with the traditional perspective: MW is a result of a lapse in attention; participants with low levels of executive function would experience high levels of MW than those with high levels of executive function. Theoretically, this model focuses on spontaneous MW, which should be experienced after the failure of the executive process^10,32^. This model supports the current results because the executive type of amotivation influenced the occurrence of spontaneous MW, which could contribute to the general association between amotivation and MW at the trait level. Thus, the key psychological component of the association between motivation and MW is executive function.

However, to be precise, dasEx is not a measurement of executive function although it is conceptually clear. Study 2 provides a more direct assessment of executive function and examines its effect on the relationship between motivation and MW using ECS, which is used to assess executive function, especially for inhibition, initiation, and attention control^22,23^, in addition to confirming the reproducibility of Study 1 in another sample of Study 2.

## Study 2

### Participants

Basically, the recruitment used for Study 2 is similar to that of Study 1. The same data collection company was used to recruit participants for Study 2; however, they were independently recruited. This study estimated that 150 participants would be included for every age group (i.e. 20–29, 30–39 and the like), resulting in 750 participants in total. Again, three *lure* items were set, where 188 participants did not follow the instructions. Finally, data from 562 participants were analysed (mean age = 45.4 ± 13.7 years; women = 263). Only certain participants (n = 35) were duplicated between the two studies, identified by the ID provided by the data collection agency.

### Measurements

Besides the measurements in Study 1, a new measurement was added to assess participants’ executive function.

#### Scale for executive function

##### Effortful control scale (ECS)

ECS is a questionnaire used to measure participants’ effortful control similar to executive function^22,23^. The questionnaire comprises 35 items, including reverse items, which were rated using a 4-point Likert-type scale. Effortful control is a single, latent temperamental construct directly linked to executive function^38^, which includes sub-components of inhibition (e.g. ‘It is easy for me to hold back my laughter in a situation where laughter wouldn’t be appropriate’ and ‘If I want to, it is usually easy for me to keep a secret’), activation (e.g. ‘I am often late for appointments’ and ‘As soon as I have decided upon a difficult plan of action, I begin to carry it out’), and attention control (e.g. ‘It is very hard for me to focus my attention when I am distressed’ and ‘When I am trying to focus my attention, I am easily distracted’). High scores indicate better executive control functions.

### Analyses

The analytical methods used in Study 1 were identical to those used in Study 2, except for the addition of another mediation model for verifying the mediating role of executive function in the relationship between motivation and spontaneous MW (i.e. independent variable = MW, mediator = ECS, and dependent variable = MW-S), which was hypothesised in Study 1. Similarly, bootstrapping (2000 samples) was used besides the conventional *z*-test to confirm the significance of the indirect effects, which does not require assuming normality of the sample distribution.

### Results

Table 2 provides the descriptive information of the results, which are illustrated in Fig. S2. Figure S4 provides the correlation coefficients among the variables.

**Figure 3:**
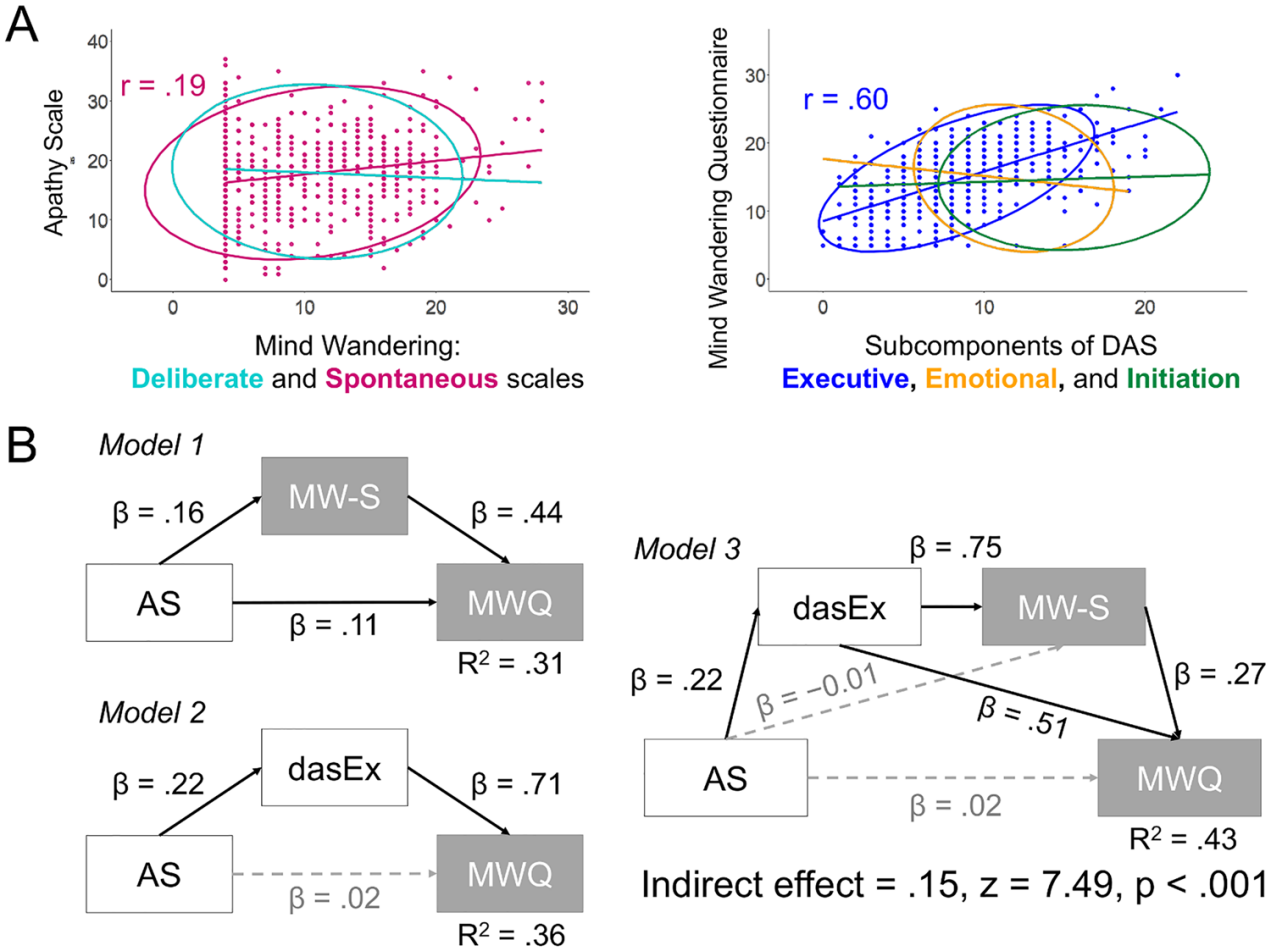
Replication of Study 1. Scatter Plots of the Correlations Between Mind Wandering and Motivational Indices (**A**) and the Results of the Mediation Analyses (**B**). *Note* As in Fig. 1, data for non-significant correlations after multiple corrections are only shown within the 95% elliptical confidence region of the multivariate t-distribution in panel **A**. In panel **B**, the models were identical as those in Study 1. The solid black line indicates the significant path, while the dashed grey line denotes the non-significant path. AS = apathy scale; MW-S = spontaneous MW scale, MWQ = mind wandering questionnaire; DAS = dimensional apathy scale and dasEx = executive subcomponent of DAS. Higher scores on the apathy scale and DAS indicate increased amotivation.

To replicate the association between motivation and MW at the trait level, this study employed correlation analysis and found that the replication was successful (AS-MWQ: *r* = 0.25 [95% CI 0.11–0.37], *p* < 0.001). Moreover, Study 1 results were also reproduced. As Fig. 3A shows, the selective significant associations between AS and MW-S (*r* = 0.19 [95% CI 0.05–0.32], *p* < 0.001) and between MWQ and dasEx (*r* = 0.60 [95% CI 0.50–0.68], *p* < 0.001) were confirmed. The remainder of the associations were non-significant (*p*s > 0.05).

Additionally, Study 2 re-conducted the mediation analyses conducted in Study 1 to confirm the replicability of Study 1 on the independent samples. As Fig. 3B shows, analyses extended the results in Study 1 through the finding that the association between AS and MWQ was entirely explained by the variance of dasEx, which indicates the possibility of executive function. The bias-corrected bootstrapped CIs for indirect effects were above 0 in all models (0.04–0.11 in Model 1, 0.11–0.20 in Model 2 and 0.11–0.20 in Model 3).

**Figure 4:**
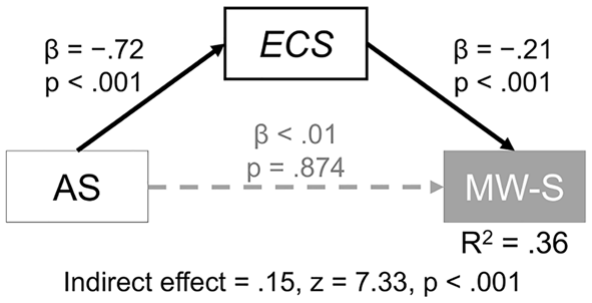
Mediation Analyses of the Relationship Between the Apathy Scale and Spontaneous Mind Wandering Scale. *Note* The ECS was the single mediator. AS = apathy scale; ECS = effortful control scale, MW-S = spontaneous MW scale.

Thus far, the study confirmed the possibility that the key component of the association between motivation and MW is executive function, which in turn causes spontaneous MW. This relationship was tested using a more direct measure of executive function (i.e. ESC). Correlation and mediation analyses revealed that ECS is more strongly associated with MW-S than MW-D (*z* = 7.83, *p* < 0.001), although these correlation coefficients were significant (ECS-MW-S: *r* = − 0.54 [95% CI − 0.59 to − 0.47], *p* < 0.001; ECS-MW-D: *r* = − 0.28 [95% CI − 0.35 to − 0.20], *p* < 0.001). Additionally, ECS mediated the relationship between AS and MW-S (Fig. 4). The bias-corrected bootstrapped CIs for indirect effect ranged from 0.11 to 0.20.

## Discussion

The present study demonstrated that the trait-level association between motivation and MW is primarily mediated by executive function, which then evokes spontaneous MW. These findings were reliable as per the replicability of the study using a different sample in Study 2.

Previous studies have reported that motivation and MW are temporally related^6,7,11^. A proposed mechanism of this state-level association indicates that individuals deliberately allow the mind to wander during unmotivated periods. In other words, individuals are expected to deliberately let their mind wander during unmotivated states in daily life. However, the current results did not support this hypothesis. The result is understandable given the dissociation between the trait and state dimensions in motivation and/or MW. Previous studies have also indicated that motivation and MW in fact share a covariance between the trait and state dimensions; however, these dimensions are weakly associated^9,13,39^. The trait–state relationship is enigmatic and is influenced by several factors, such as memory, metacognitive ability and time or season of the test. The current study provided an insight into understanding the divergence between the trait and state dimensions. Contrary to the hypothesis that amotivation induces deliberate MW, this study found that the lower the level of motivation, the more the mind wanders in a spontaneous fashion at the trait level, whose association depends on executive function.

The concept of the subdivision of motivation in the current study emerged from the perspective of apathy^17,18,40^. Although the importance of the subdivision in healthy individuals remains unclear, the executive component may be the most important factor of apathy in patients without dementia^41,42^ and healthy adults^19,20^. In this study, the subdivision suggested that executive dysfunction may be the mechanism that underlies the association between motivation and MW, which could lead to spontaneous MW at the trait level. Using a more direct assessment of executive function can corroborate this finding. As noted in the Interim Discussion section, the executive failure hypothesis of MW supported the results. Individuals with high levels of executive capacity reported more on-task and less off-task thoughts during experimental tasks compared to those with low levels of executive capacity^3,6,14,15^. Based on such a clear relationship, this hypothesis suggests that the inability to focus on a task and task-relevant goals could result in MW. Specifically, the experience sampling method found that participants with low levels of executive function experienced high rates of MW in daily life in terms of the effect of executive function on MW at the trait level^43^. This finding is consistent with the current results. Conversely, several studies have proposed the link between high levels of cognitive capacity and increased MW^4,44^. This seemingly contrasting phenomenon, explained by executive failure and executive control accounts, are perhaps dependent on a certain context, that is, the demands of the task^5,45^ (see study^46^ for another reconciliation). People with high levels of executive skills could adaptively select their mental activities from avoiding MW that leads to deleterious consequences during demanding tasks^6,15^ to allowing their mind to wander during non-demanding tasks^4,14,44^. In the latter case, studies have confirmed the adaptive aspect because people could consider the future^47^ or think in a creative manner^48^. Based on this account of context regulation hypothesis^45^, the current study suggests that the introspective nature of a questionnaire survey weights or biases MW during specific tasks in daily life with relatively high demands. As an interpretation, people tend to remember these MW more clearly than the MW during tedious tasks or no task because such MW during tasks in high demands would result in negative consequences. Perhaps this introspective and/or memory bias could explain the absence of correlation between deliberate MW and trait motivation, although there are other possibilities (e.g. people with less motivation at the trait level may lack the motivation to let their mind wander deliberately because deliberate MW needs a cognitive load to a certain extent). Because the specific measures used here cannot support or deny this hypothesis, future research may need to adopt measurements for the contexts that individuals are in when they are MW so as to consider the inference of an introspective bias.

The potential limitations of this study are as follows. First, the causality included in the mediation analyses seems inappropriate because the causal effect of amotivation on executive function may be theoretically questionable, especially for the model shown in Fig. 4. The result should be interpreted as follows: the covariance between executive function and amotivation evokes spontaneous MW and diverts attention from the causal relationship. Second, the application of the apathy subdivision to motivation in healthy individuals may be controversial. Although apathy is clearly a disorder of motivation^16^ several apathy scales had been initially developed by employing healthy populations^18,29,40^, one can claim that this application is inadequate. Moreover, the reliability of dasEm was insufficient in both Studies 1 and 2, which was also demonstrated previously^29^. Further investigation of these points may be required. Third, the retrospective method may bias the results. Besides the aforementioned nuisance effect of memory, such self-reported data would be contaminated with the mood and neurocognitive state during the survey^5^; moreover, trait-state and survey test inconsistency were observed in MW^13,49^. Moreover, studies on executive function have demonstrated such a discrepancy^50^. To draw solid conclusions, an experimental study that utilises task-based assessments for MW (i.e. experience sampling method) and executive function (i.e. Stroop’s task and N-back task) are warranted. Fourth, the characteristics and conditions of the participants were totally unknown, except for age and sex. The study did not conduct screening for cognitive impairment.

Irrespective of these limitations, the current findings can provide meaningful insights for understanding motivation and MW at the trait level and aid in developing interventions to control these tendencies in daily life. Although this study used the correlational approach, its adequate sample size and self-replication provided a solid conclusion that the failure of executive function can explain the association between motivation and MW at the trait level, which may be biased by the use of retrospective methods.

### Ethical approval

All procedures performed in the studies involving human participants were conducted in accordance with the ethical standards of the Institutional Review Board with the 1964 Helsinki Declaration and its later amendments or comparable ethical standards. Informed consent was obtained from all participants.

## Supplementary Information

Supplementary Information.

## Data Availability

The datasets collected and/or analysed in this study are not publicly available because a joint research agreement is required for data sharing. However, information is available from the corresponding author upon reasonable request.
