# Supplementary material for: Executive failure hypothesis explains the trait-level association between motivation and mind wandering
Source: Sci Rep. 2022 Apr 7;12:5839. doi: 10.1038/s41598-022-09824-3 (PMC8990005; doi:10.1038/s41598-022-09824-3)
Supplement: Supplementary file 1 — Supplementary Information. [file 41598_2022_9824_MOESM1_ESM.docx]

**Supplementary information**

**Supplementary Results**

***Distribution of data***

Figures S1 and S2 display the data distribution in Studies 1 and 2, respectively.

**Figure S1** *Distributions of measurements in Study 1*

******

**Figure S2** *Distributions of measurements in Study 2*

***Distribution of data***

Figures S3 and S4 display the correlation coefficients among the variables in Studies 1 and 2, respectively.

***
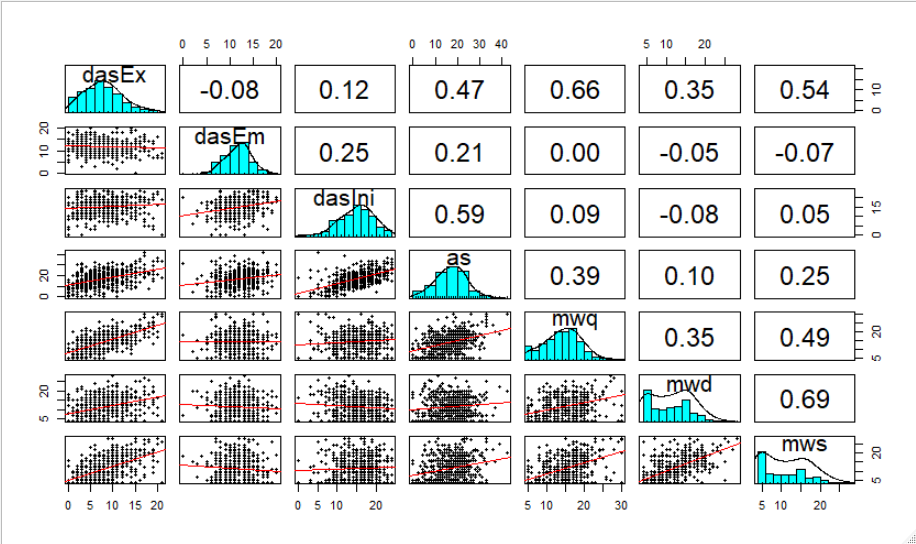
***

**Figure S3** *Correlation coefficients in Study 1*

*
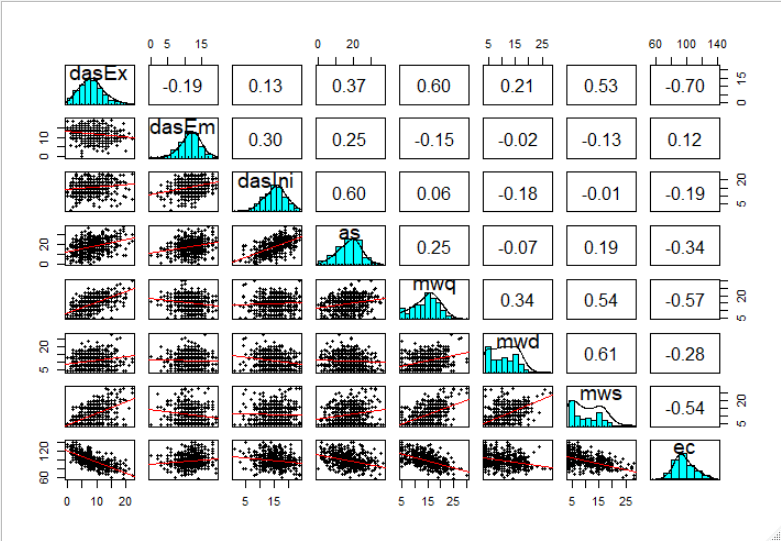
*

**Figure S4** *Correlation coefficients in Study 2*

***Non-parametric correlational analysis***

Study 1

Since normality was seemingly violated in the MW-D and MW-S, non-parametric correlational tests were applied to the correlations, which includes these variables. Spearman’s correlation coefficients between AS and MW-D/S were significant (MW-D: *r* = 0.13 [95% CI: 0.05–0.21], *p* = 0.031; MW-S: *r* = 0.27 [95% CI: 0.19–0.34], *p* < 0.001). However, the difference was significant, as in the case of parametric tests (*z* = 4.53, *p* < 0.001).

Study 2

Spearman’s correlation coefficients between AS and MW-S was again significant (*r* = 0.18 [95% CI: 0.09–0.25], *p* < 0.001), whereas that between AS and MW-D was non-significant (*r* = −0.05 [95% CI: −0.13–0.03], *p* = 0.212). Their difference was also significant (*z* = 6.57, *p* < 0.001).
